# Socioeconomic position and the effect of price interventions on healthier and less healthy food on food selection in a simulated food delivery app: a randomised controlled trial

**DOI:** 10.1017/S1368980026103334

**Published:** 2026-08-10

**Authors:** Rozemarijn Witkam, Jane Brealey, Loukia Tzavella, Amy Finlay, Andrew Jones, Eric Robinson

**Affiliations:** 1 Division of Musculoskeletal & Dermatological Sciences, Faculty of Biology, Medicine & Health, School of Biological Sciences, The University of Manchester, UK; 2 Department of Psychology, Institute of Population Health, University of Liverpool, UK; 3 School of Psychology, Liverpool John Moores University, UK

**Keywords:** Obesity, Fiscal policy, Out-of-home food sector, Simulation study

## Abstract

**Objective:**

We examined the effects of price interventions on the healthiness of a hypothetical food order in a simulated online out-of-home food delivery app.

**Design:**

The design was a randomised controlled trial, with four conditions (10 % taxation of less healthy menu items; 10 % subsidy of healthier menu items; a combination of taxation and subsidy; standard pricing), and recruitment was stratified by socioeconomic position (SEP). Primary outcomes were healthier *v*. less healthy main meal selected and total kilocalories (kcals) selected.

**Setting:**

Participants selected a hypothetical evening meal from out-of-home food outlets on a simulated online food delivery app.

**Participants:**

*N* 3106 adult participants from the UK were randomised into taxation (*n* 750), subsidies (*n* 808), a combination of taxation and subsidies (*n* 769), and standard pricing (*n* 779). 57–58 % of participants were of higher SEP.

**Results:**

There were no statistically significant effects of the pricing interventions on whether a healthy *v*. less healthy main meal was selected (e.g. taxation *v*. control OR 0·91 (95 % CI 0·74, 1·12); subsidies *v*. control OR 1·08 (95 % CI 0·89, 1·31)) or on total kcals selected (e.g. taxation *v*. control regression coefficient −44·31 (95 % CI −95·90, 7·28); subsidies *v*. control regression coefficient −7·94 (95 % CI −60·23, 44·35)). There was no evidence that effects differed by SEP.

**Conclusions:**

We found no convincing evidence that 10 % changes in price of healthy and less healthy food in a simulated out-of-home food delivery app significantly impacted on healthiness of food selected. Further research may benefit from examining effects of larger price changes and in real-world purchasing conditions.

Diets high in energy, saturated fat, salt and sugar are a major contributor to obesity, non-communicable diseases (such as CVD, diabetes and cancer)^(1)^ and premature death^(2)^. Poor diet and obesity are more common in those with lower socioeconomic position (SEP), and this likely contributes to socioeconomic inequalities in health^(1–3)^.

Food prepared out of the home, such as in restaurants and takeaway meals, tends to be of low nutritional quality and energy dense^(4,5)^. Frequent use of the out-of-home food sector (OHFS) is associated with obesity^(6)^. Specifically, the use of online food delivery platforms has increased in the UK in recent years and surged during the COVID pandemic^(7)^. After China and the USA, the UK is the third biggest online food delivery market in the world^(8)^. A 2024 cross-sectional study of survey and consumer data in England found that those with lower SEP were more likely to use food delivery apps (OR 2·3 (95 % CI 1·38, 3·87)) compared to those with higher SEP^(9)^. In this study, the use of food delivery apps was also associated with increased obesity risk (relative risk ratio 1·8 (95 % CI 1·2, 2·8))^(9)^.

In a 2024 report, the WHO recommended that governments should adopt fiscal policies to improve population health and combat diet-related diseases^(10)^. Food affordability, which is determined by food prices and disposable income, is a crucial component of the food environment and is known to have a significant impact on food purchases^(11)^. Taxes and subsidies can therefore be used to change food purchasing. Fiscal policies may also have the potential to reduce inequalities in diet as it is thought that people from lower SEP backgrounds are more motivated by price^(12)^ and less motivated by health or weight control^(13)^ compared to higher SEP backgrounds. Moreover, taxation may encourage food manufacturers to reformulate their products to be healthier^(14)^.

A systematic review and meta-analysis including twenty-three intervention studies and seven prospective studies in a variety of settings (i.e. supermarkets, schools, cafeterias), reported that a standardised 10 % decrease in price (i.e. subsidy) increased consumption of healthier products by 12 % and a standardised 10 % increase in price (i.e. tax) decreased consumption of less healthy products by 6 %^(15)^. However, many of the included subsidy studies incorporated other intervention components, such as promotion campaigns advertising the healthier foods and price decreases, which may explain some of the greater effect of subsidies *v*. taxation. Because of this, intervention studies directly comparing subsidies and taxation as methods for changing diet would be valuable. Specifically, there is a lack of research comparing the effects of fiscal interventions in the context of food delivery apps^(16)^. Given the growing importance of food delivery apps to national diet, understanding of policy options to improve healthiness of diet on these platforms will be important.

Most fiscal policies that have been studied or implemented in the real world have focused on specific food groups, such as taxes on sugar-sweetened beverages or subsidies on fruit and vegetables^(15)^. However, research indicates that in order to lower the risk of obesity and other diet-related diseases, it is preferable to focus on overall dietary patterns rather than specific food groups^(17)^. One way to quantify nutrient quality of foods is using the UK Nutrient Profiling Model (NPM)^(18)^. The UK NPM categorises foods as ‘healthier’ and ‘less healthy’ based on the proportion of nutrients that people should generally consume less of (saturated fat, sugar and salt) with that of nutrients needed for a healthy diet (including protein, fibre, fruit, vegetables and nuts). The model has been shown to be in line with national dietary guidelines (e.g. the Eatwell Guide recommended by the National Health Service in the UK)^(19)^, and higher consumption of foods categorised as less healthy is linked with weight gain, non-communicable diseases and mortality^(20–24)^.

Most trials to date which have studied the effect of food taxation and subsidy on food selection have used relatively large price changes of 20–50 %^(15,25)^, which do not reflect the size of price change that fiscal policies in the real-world typically result in. For example, a recent report on the implementation of taxation of sugar-sweetened beverages worldwide reported that the effective tax rate for a 330 ml sugar-sweetened carbonated beverage was only 6·6 %^(26)^. Examining more realistic price changes is therefore important for understanding likely policy impacts of food taxation or subsidies.

In the present study, we used a randomised controlled trial to examine effects of 10 % price increases on less healthy food (taxation), 10 % price decreases on healthier foods (subsidies) and a combination of taxation and subsidy relative to a control condition (no price changes) in a simulated UK food delivery app. We also compared the taxation and subsidy condition directly to understand which has a greater influence on healthy eating behaviour when implemented separately. We further examined whether impacts of interventions on food choice differed by participant SEP through the use of stratified sampling. This approach allowed us to understand likely social equity of pricing interventions in the out-of-home food ordering sector.

## Methods

### Design

The design was an randomised controlled trial, with 4 conditions (10 % taxation of less healthy menu items; 10 % subsidy of healthier menu items; a combination of taxation and subsidy; standard pricing) × 2 (high *v*. low SEP) between-subject conditions. The study was double-blinded (i.e. both the participants and researchers were not aware which intervention group participants were assigned to).

### Participants and recruitment

Participants were recruited via Prolific, which is a global research platform (www.prolific.com), between 28^th^ June 2024 and 1^st^ August 2024. Using Prolific’s pre-screening feature, participants were invited to participate if they were a UK resident and aged 18 years and older. Participants were told it was a study about understanding food choices when using a food delivery app; they were not aware of the exact study aims prior to participating.

We stratified recruitment by gender (approximately 50 % male and 50 % female) and SEP, defined by participants’ household income. Based on the 2022 reported median yearly household income after tax in the UK of £32,300^(27)^, we considered household incomes of £29 999 and below as lower SEP and incomes of £30 000 and above as higher SEP. After stratified recruitment of participants into four subgroups (i.e. lower SEP males; lower SEP females; higher SEP males; higher SEP females), participants were randomly 1:1 allocated in one of the four conditions using a code in Inquisit (see section Simulated food delivery app).

On completion, participants were paid proportionately for the average time taken to complete the study (equivalent to £9 per hour).

### Interventions

There were three intervention groups (10 % taxation of less healthy menu items; 10 % subsidy of healthier menu items; and a combination of taxation and subsidy) and one control group (standard pricing).

Price reductions or increases were based on whether the dish would be classified as ‘healthier’ or ‘less healthy’ by the UK NPM^(18)^. Detailed information on the scoring system is provided in the supplementary materials (Box S1, Table S1, Table S2). In short, it gives scores to three healthier components per 100 grams of a food item (fruit, vegetables and nuts; fibre; protein) and to the four ‘less healthy’ components per 100 grams (energy; saturated fat; total sugar; sodium). An overall score is then calculated by deducting the healthier components from the less healthy components. The NPM calculator (https://npmcalculator.cdrc.ac.uk/), developed by researchers at the University of Leeds, was used to calculate the NPM scores of the dishes. Nutritional information of each dish was taken from the websites of the food outlets. Whilst NPM scores are useful for characterising food item nutrient quality, the NPM was not originally developed for use in the OHFS. For example, NPM does not account for portion size and energy content. For the purpose of the present study and application of the NPM to the OHFS, we modified the NPM. If a main dish contained an excessive number of kcals (i.e. 1000 kcal or more^(4)^), it was categorised as less healthy. Similarly, sides and desserts were categorised as less healthy if they contained 600 kcal or more (based on Public Health England’s guidelines for a full main meal^(28)^ and the definition of excessive kcal content for sides and desserts by Muc et al.^(5)^). Whilst this approach had good face validity, when applied to OHFS foods we noted that NPM could result in a menu item containing more than an adult’s total daily recommended nutrient intake (e.g. >6 grams of salt per day^(29)^). In a small number of instances where this was observed, we downgraded menu items to ‘less healthy’.

#### Simulated food delivery app

A simulated food delivery app was developed using the programme Inquisit6^(30)^. The interface was modelled on a popular UK-based food delivery platform, using publicly available menu information from four large restaurants: a Mexican fast-food chain, a healthier on-the-go fast-food chain, a pizza restaurant chain and a Japanese restaurant chain. For each selected restaurant, we extracted item names, descriptions, prices and photographs of mains, sides, desserts and non-alcoholic drinks directly from the platform’s website and imported these into Inquisit at the time the app was created (April 2024). More information is provided in Section 2·4.

The four food outlets used provided different ratios of healthier *v*. less healthy dishes and a range of different priced menu items (see Supplementary Material, Tables S3, S4 and S5). We opted to allow participants to choose a meal from one of four food outlets as we reasoned that implementation of price changes could result in switching behaviour (e.g. favouring a different vendor to purchase a meal from), and this may be dependent on price points of the food outlets.

### Procedures

Prolific panel members aligning with inclusion criteria were invited to participate. Participants were redirected to Inquisit, where the mock delivery app was hosted, and randomised.

After providing informed consent, participants completed a baseline questionnaire about their demographic characteristics. Next, participants completed the mock delivery app task and were asked to order an evening meal for themselves from one of the four outlets. Specifically, the instructions stated:

*‘We would like you to imagine that you are going to order and pay for an evening meal for yourself from a food delivery app. On the next page, you will see a simulated food delivery app with four different food outlets to order from. You can browse between outlets, but you can only order from ONE outlet. Please order a main meal and you have the option of including any sides, desserts or drinks in your meal order. Then you can complete your order by clicking the “basket” symbol in the top right corner. Please imagine this is a real-life scenario, where you will pay for the meal yourself and eat the meal for dinner tonight’.*

Participants were able to move back and forward through the different outlets and through menu pages for each outlet using the ‘next page’, ‘previous page’ and ‘Homepage’ buttons. The Supplementary Materials (Box S2–S6) show screenshots of what the app looked like. When participants clicked on the basket, to complete their food selection, another message was shown: ‘*Are you sure you have finished your selections? Remember: you need to order a main meal and you have the option of including any sides, desserts or drinks in your meal order. Once you are inside the basket, you will NOT be able to go back to the menu to add additional items!’.* In the basket (Supplementary Materials, Box S7), participants were then asked to either ‘order’ the item(s), which meant that they had completed the food order task and were redirected to the follow-up questionnaire, or ‘remove’ the item(s), which would bring them back to the main page showing the four food outlets and they could start their food order task again.

Presentation was reproduced from the respective food outlet pages on a popular food delivery app. Menu items were displayed with titles, pictures, prices (dependent on which condition) and the number of kcal per serving. The text ‘Adults need around 2000 kcal a day’ was included on each page (as per the current kcal labelling legislation^(31,32)^). Bundle meal deals were not made available on the app as combinations of bundle options could not be reliably categorised as less healthy *v*. healthier (e.g. in instances where bundles included both less healthy and healthier menu options).

In the follow-up questionnaire, participants were asked to guess the aim of the study. They were further asked about whether they thought the mock delivery app was representative of existing delivery apps and their perception of taxation and subsidies as a measure to improve healthy eating. Finally, participants were debriefed about the study aims.

### Variables

#### Primary outcome measures

Primary outcomes were healthier *v*. less healthy main meal selected (binary) and total kcal of all food selected (continuous).

#### Secondary outcome measures

As participants could also (optionally) select additional sides and desserts, the proportion of healthier *v*. less healthy meal items chosen from the total food order was examined. For instance, if participants selected a healthier main, but a less healthy side and dessert, the proportion of healthier meals was 0·33.

To examine nutrients selected, total fat, saturated fat, salt and sugar were calculated based on the total food order.

Total money spent (in £) was also examined.

#### Participants characteristics and socioeconomic variables

In the baseline questionnaire, we asked participants to report their age (continuous, years), sex (categorical – male, female, other, prefer not to answer), ethnic group (categorical – White; Black/African/Caribbean/Black British; Asian/Asian British; Mixed/Multiple ethnic groups; Other; prefer not to answer), height (continuous, metres or in feet) and weight (continuous, kg or in stone). BMI was then calculated by dividing the weight (in kg) by the height (in metres(m)^2^). The WHO cut-off points were used to define underweight (BMI < 18·5 kg/m^2^), normal weight (BMI 18·5–24·9 kg/m^2^), overweight (BMI 25·0–29·9 kg/m^2^) and obesity (BMI ≥ 30·0 kg/m^2^).

Participants reported on a range of socioeconomic variables, including household income, food security, education, employment status and subjective SEP.

Equivalised net monthly household income was estimated by dividing the midpoint of monthly household income after tax (categorical – Under £800; £800–£1500; £1600–£2300; £2400–£3100; £3200–£3900; £4000–£4700; £4800–£5500; £5600–£6300; £6400–£7100; £7200 or more, Prefer not to answer) by the weighting category assigned dependant on the number of household members as recommended by Kuhn (2019)^(33)^. The first adult received a weighting of 1·0, the second adult and any persons aged 14 years and older a weighting of 0·5 and children younger than 14 years a weighting of 0·3. To enable stratified analysis, this variable was dichotomised into lower monthly equivalised household income (i.e. £1500 or less) and higher monthly equivalised household income (i.e. more than £1500). The cut-off point of £1500 was selected because it is less than 60 % of medium UK household net income^(34)^.

Food security was measured using the household food security scale as described in the annual UK Family Resources Survey 2021^(35)^. The scale measures whether people have had access to adequate food to maintain an active and healthy lifestyle in the past month. Households are then categorised into four groups: high food security (score = 0), marginal food security (score = 1 or 2), low food security (score = 3–5) and very low food security (score = 6–10)^(35)^. This variable was dichotomised into higher food security (i.e. high and marginal food security) and lower food security (i.e. low and very low food security).

Highest educational qualification was coded from less than high school (1); high school completion (2); some college or associate degree (3); bachelor’s degree (4); master’s degree (5); doctoral degree (6). For the purpose of analyses, education was dichotomised into lower education (categories 1–3) and higher education (categories 4–6).

Subjective socioeconomic status was measured using the MacArthur Scale^(36)^, which measures people’s perceived socioeconomic status based on money, education and jobs compared to others. Participants were asked to place themselves on a ladder (1–10; 1 = lowest and 10 = highest subjective social status).

#### Other variables

In the follow-up questionnaire after completing the food ordering task, we asked participants ‘what do you think the aim of this study is?’. If participants were able to link food prices with food selection, they were considered as having guessed the aims of the study.

Participants were then asked whether they thought the mock delivery app was representative of existing food delivery apps on a 7-point Likert Scale, from strongly disagree to strongly agree.

To understand participants’ attitudes towards fiscal policy interventions in the OHFS, we questioned to what extent they agreed with (i) taxation of less healthy dishes and (ii) subsidies on healthier dishes to encourage healthy eating. Similarly, a 7-point Likert Scale was used from strongly disagree to strongly agree.

### Analyses

The analysis plan for this study was pre-registered (https://osf.io/ahx8m and https://clinicaltrials.gov/study/NCT06468501)

#### Primary outcomes

Binary logistic regression examined the individual effects of each pricing intervention compared to the control condition (independent variable) on healthier *v*. less healthy main meal choice (dependent variable). We also directly compared taxation and subsidies in a binary regression analysis. These analyses were repeated on the primary outcome of total kcal ordered (linear as opposed to logistic regression).

To understand whether SEP moderated the impact of the interventions on the primary outcomes, an interaction term was added between each intervention (i.e. taxation *v*. control; subsidies *v*. control; and a combination of taxation and subsidies *v*. control, binary variables) and SEP.

#### Secondary outcomes

The primary analyses were repeated on the secondary outcomes using Poisson regression analysis for the proportion of healthier meal items *v*. less healthy meal items and linear regression analysis for total sugar, fat and salt intake (continuous). To account for multiple comparisons, the *p*-value for the secondary outcomes were adjusted using the Bonferroni correction method (*p* < 0·01).

#### Analysis of follow-up questionnaire

Descriptive analyses were used to understand the extent that participants thought the mock delivery app was representative of existing food delivery apps, and what participants’ perceptions were towards fiscal policies to encourage healthier eating in the OHFS.

#### Sensitivity analyses

To ensure robustness of the findings, primary analyses were repeated when participants with dietary restrictions/food allergies were excluded and when participants who guessed the study aim were excluded.

Furthermore, interaction effects of other measures of SEP (household food security, education and subjective socioeconomic status) and demographic factors (sex, BMI, ethnic group) were estimated on the primary outcome measures. The *p*-value for the interaction effects were adjusted (*p* < 0·01) to account for multiple comparisons.

All analyses were performed using Stata V18.

#### Sample size calculation

G*power was used to perform an a-priori sample size calculation for the primary analyses. Based on a previous meta-analysis, we estimated 779 participants were needed in each condition to detect a small effect (OR 1·33 equivalent to Cohen’s *f* of 0·08), with 80 % power and an error probability of 5 %. This resulted in a required total target sample size of *n* 3116. Taking into account attrition and uncertainty of expected effects, we aimed to recruit *n* 3464 (*n* 866 per condition). Although we recruited the required number of participants (*n* 3514), only *n* 3106 were randomised into the four conditions (see Result section for reasons). This means that we were slightly under the desired power threshold. For full sample size calculation information, see the online supplementary materials, Box S8.

## Results

### Data exclusions and sample characteristics

**Figure 1.**
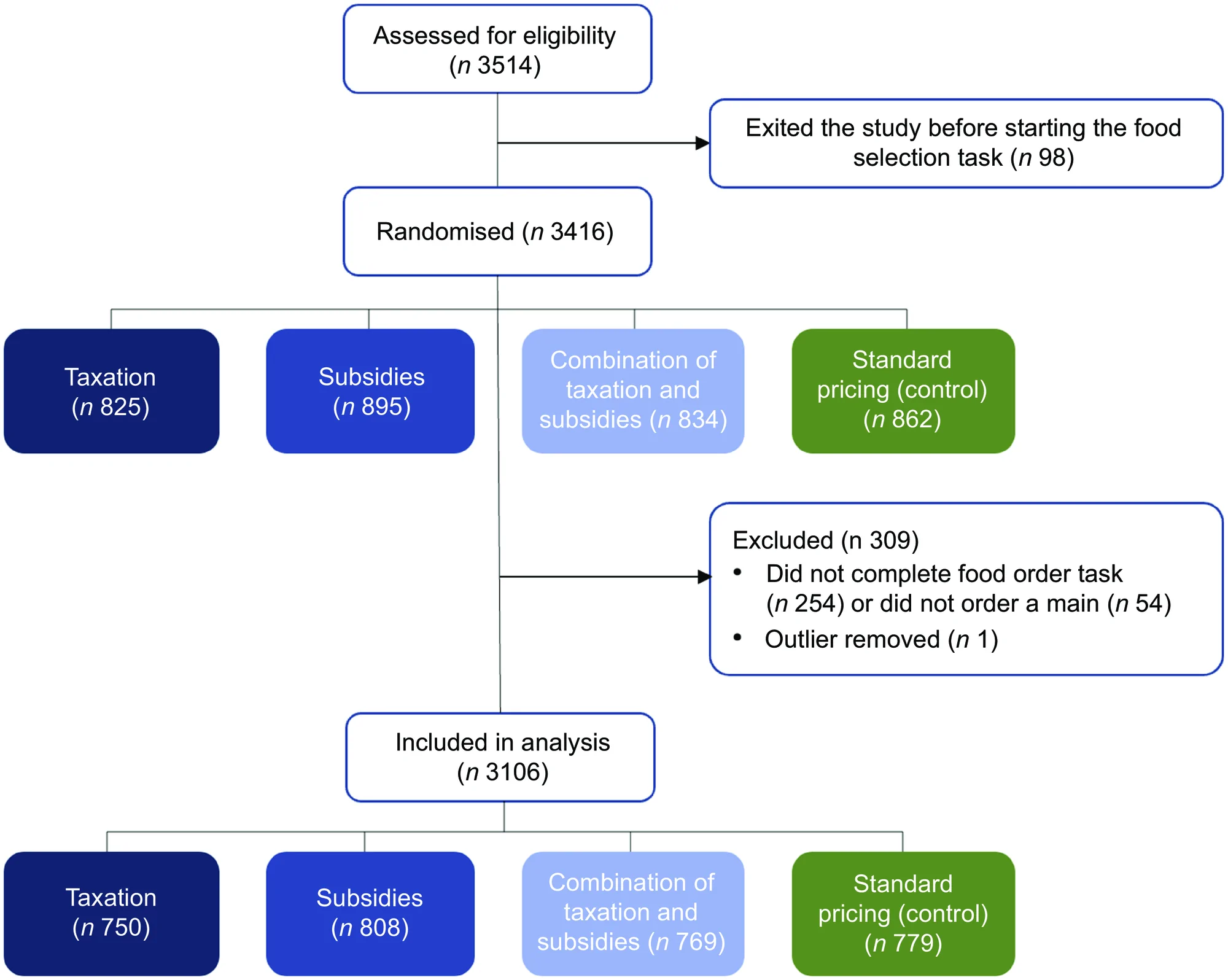
CONSORT participant flow diagram.

Of the 3514 participants who started the study, *n* 98 exited the study before randomisation. *N* 3416 participants were randomised into the four conditions. Of these, *n* 309 were excluded due to not completing the food ordering task (*n* 254), not ordering a main meal (*n* 54) or being an outlier in the number of kcals ordered (i.e. higher than a *z*-score of 3) (*n* 1). In instances where participants made minor ordering errors, we recoded data accordingly (see Supplementary Materials, Box S9). In total, *n* 3106 were included in the analyses, divided into taxation (*n* 750), subsidies (*n* 808), a combination of taxation and subsidies (*n* 769), and standard pricing (control) (*n* 779) (Figure 1).

Sample characteristics were similar in each condition (Table 1). In each group, mean age was approximately 37 years, approximately 50 % of participants were female and 75 % were of White ethnicity. A slightly higher proportion of the participants had a higher income status (between 57 % and 58 % in each condition) compared to a lower income status.

**Table 1.** Descriptive statistics of the total sample (*n* 3106) by condition Table 1 long description.

	Taxation (*n* 750)		Subsidies (*n* 808)		Combination (*n* 769)		Standard pricing (*n* 779)	
	*n*	%	*n*	%	*n*	%	*n*	%
Sample characteristics								
Age, years								
Mean	37·0		37·3		36·9		36·7	
sd	12·6		12·7		12·8		12·9	
Sex, female	369	49·2%	415	51·4%	396	51·5%	368	47·2%
Ethnicity, White	566	75·9%	604	74·9%	579	75·4%	586	75·3%
*Missing*	*4*	*0·5%*	*2*	*0·2%*	*1*	*0·1%*	*1*	*0·1%*
Equivalised net monthly household income, £								
Mean	1869·2		1921·8		1907·5		1903·6	
sd	1097·7		1126·4		1129·9		1126·5	
Higher income status^*^	417	57·1%	456	58·2%	425	56·7%	437	58·4%
*Missing*	*20*	*2·7%*	*25*	*3·1%*	*19*	*2·5%*	*31*	*4·0%*
Higher food security†	579	77·2%	635	78·6%	586	76·2%	607	77·9%
Education, bachelor’s degree or higher	459	61·2%	484	59·9%	490	63·7%	483	62·0%
Subjective social status, 0–10 (10 = highest)								
Mean	5·2		5·3		5·2		5·2	
sd	1·5		1·6		1·6		1·6	
*Missing*	*1*	*0·1%*	*0*	*0·0%*	*1*	*0·1%*	*1*	*0·1%*
Employment status, full-time or part-time	567	75·6%	603	74·6%	566	73·6%	568	72·9%
Employment status, student	69	9·2%	69	8·5%	71	9·2%	83	10·7%
Employment status, retired or other	114	15·2%	136	16·8%	132	17·2%	128	16·4%
BMI, kg/m^2^								
Mean	27·1		27·6		26·9		26·9	
sd	6·5		6·8		6·1		6·5	
Overweight or obesity (BMI ≥ 25)	422	56·3%	473	58·5%	433	56·3%	417	53·5%
*Missing*	*4*	*0·5%*	*4*	*0·5%*	*3*	*0·4%*	*2*	*0·3%*
Outcome variables								
Healthier main meal ordered, yes	314	41·9%	372	46·0%	342	44·5%	344	44·2%
Total kcal ordered								
Mean	1264·8		1301·2		1287·7		1309·1	
sd	487·7		523·6		521·4		538·4	
Proportion of items selected that were healthier								
Mean	0·38		0·39		0·39		0·38	
sd	0·37		0·38		0·38		0·37	
Total fat ordered, g								
Mean	51·9		53·6		53·4		54·0	
sd	25·3		25·2		26·4		26·8	
Total saturated fat ordered, g								
Mean	15·9		16·2		16·4		16·2	
sd	10·4		10·7		11·1		10·6	
Total salt ordered, g								
Mean	6·5		6·7		6·5		6·6	
sd	3·0		3·4		3·4		3·4	
Total sugar ordered, g								
Mean	29·3		29·5		29·8		29·8	
sd	23·6		23·9		23·1		22·9	
Total spend, £								
Mean	22·3		21·3		21·8		21·9	
sd	9·7		10·0		10·3		10·1	
Other variables								
Guessed aim of the study, yes	57	7·6%	67	8·3%	61	7·9%	59	7·6%

* Defined as a monthly net household income of higher than £1500. The percentage is calculated from those without missing income data. †Defined as a high or marginal food security (scores 0–2). g, grams.

### The effect of the pricing interventions on primary and secondary outcomes

There were no significant effects of any of the pricing interventions on whether a healthy *v*. less healthy main meal was selected (Table 2). There were some small directional effects of the interventions on total kcal selected, whereby participants in the taxation condition selected descriptively fewer kcals (3 %) than the control condition. However, these differences were not statistically significant (Table 2) and were small in size.

**Table 2.** The effect of the pricing interventions (taxation, subsidies and a combination of taxation and subsidies) on primary outcomes in total sample (*n* 3106) Table 2 long description.

Healthier v. less healthy main meal selected			
Conditions	OR	95 % CI	*p*-value
Taxation *v*. control	0·91	0·74, 1·12	0·37
Subsidies *v*. control	1·08	0·89, 1·31	0·45
Combination *v*. control	1·01	0·83, 1·24	0·90
Taxation *v*. subsidies	0·84	0·69, 1·03	0·10

Total kcal selected			
Conditions	Regression coefficient	95 % CI	*p*-value
Taxation *v*. control	−44·31	−95·90, 7·28	0·09
Subsidies *v*. control	−7·94	−60·23, 44·35	0·77
Combination *v*. control	−21·42	−74·27, 31·43	0·43
Taxation *v*. subsidies	−36·37	−86·76, 14·02	0·16

There were no statistically significant effects of the interventions on the secondary outcomes (Table 3), including proportion of healthier *v*. less healthy items selected and nutrients selected, and no evidence that effects of pricing interventions differed by SEP (household income). See Supplementary materials, Tables S6 and S7.

**Table 3.** The effect of the pricing interventions (taxation, subsidies and a combination of taxation and subsidies) on secondary outcomes in final sample (*n* 3106) Table 3 long description.

Conditions	Regression coefficient	95 % CI	*p*-value
**Proportion of items selected that were healthier**			
Taxation *v*. control	−0·00	–0·17, 0·16	0·98
Subsidies *v*. control	0·04	−0·12, 0·20	0·65
Combination *v*. control	0·03	−0·13, 0·19	0·70
Taxation *v*. subsidies	−0·04	−0·20, 0·12	0·63
**Total sugar selected**			
Taxation *v*. control	−0·53	−2·86, 1·80	0·66
Subsidies *v*. control	−0·36	−2·67, 1·94	0·76
Combination *v*. control	−0·02	−2·31, 2·28	0·99
Taxation *v*. subsidies	−0·16	−2·52, 2·19	0·89
**Total fat selected**			
Taxation *v*. control	−2·06	−4·67, 0·56	0·12
Subsidies *v*. control	−0·37	−2·94, 2·19	0·78
Combination *v*. control	−0·56	−3·21, 2·09	0·68
Taxation *v*. subsidies	−1·69	−4·20, 0·83	0·19
**Total saturated fat selected**			
Taxation *v*. control	−0·30	−1·35, 0·75	0·58
Subsidies *v*. control	0·02	−1·03, 1·07	0·97
Combination *v*. control	0·17	−0·91, 1·24	0·76
Taxation *v*. subsidies	−0·32	−1·37, 0·73	0·55
**Total salt selected**			
Taxation *v*. control	−0·11	−0·43, 0·22	0·52
Subsidies *v*. control	0·06	−0·28, 0·39	0·74
Combination *v*. control	−0·14	−0·47, 0·20	0·43
Taxation *v*. subsidies	−0·16	−0·48, 0·16	0·32
**Total spend**			
Taxation *v*. control	0·37	−0·63, 1·36	0·47
Subsidies *v*. control	−0·63	−1·62, 0·36	0·21
Combination *v*. control	−0·07	−1·09, 0·95	0·90
Taxation *v*. subsidies	1·00	0·02, 1·98	0·05

### Follow-up questionnaire

Of the participants that completed the follow-up questionnaire (*n* 3066), most agreed (from slightly agree to strongly agree) that the app was representative of existing food apps (80·9 %, *n* 2481) (Table 4).

**Table 4.** Descriptive analysis of follow-up questionnaire in final sample (*n* 3066) Table 4 long description.

Question	Strongly disagree		Disagree		Slightly disagree		Neither agree or disagree		Slightly agree		Agree		Strongly agree	
	*n*	%	*n*	%	*n*	%	*n*	%	*n*	%	*n*	%	*n*	%
**I felt that the app was representative of existing food apps**														
	63	2·1	115	3·8	219	7·1	188	6·1	833	27·2	1240	40·4	408	13·3
**To what extent do you agree with the government charging out-of-home food businesses more tax when they sell unhealthy dishes to help people make healthier choices?**														
	374	12·2	525	17·1	460	15·0	505	16·5	677	22·1	378	12·3	147	4·8
**To what extent do you agree with the government charging out-of-home food businesses less tax when they sell healthier dishes to help people make healthier choices?**														
	160	5·2	220	7·2	203	6·6	537	17·5	802	26·2	730	23·8	414	13·5
To what extent do you agree with the government implementing a combination of taxing businesses more for selling unhealthy dishes whilst taxing businesses less for selling healthier dishes in the out-of-home food sector to help people make healthier choices?^*^														
	219	7·2	323	10·5	308	10·1	742	24·2	758	24·7	477	15·6	238	7·8

* Sample of *n* 3065.

Participants were more in favour of subsidies (63·5 % slightly to strongly agreed) than taxation (39·2 % slightly to strongly agreed) or a combination of taxation and subsidies (48·1 % slightly to strongly agreed) as policies in the OHFS(Table 4).

### Sensitivity analyses

Primary analyses were repeated when participants with dietary restrictions/food allergies were excluded (*n* 2705) and when participants who guessed the study aim were excluded (*n* 2863). Results did not differ from the main study sample (supplementary materials, Tables S8 and S9).

There were no interaction effects between any demographic characteristics and intervention condition for outcomes (supplementary materials, Table S10).

## Discussion

### Summary of results

The aim of this randomised controlled trial was to study the effects of pricing interventions based on menu item healthiness (i.e. a 10 % price difference) by comparing the effects on food selection in scenarios of taxation (i.e. increasing price of less healthy items), subsidies (i.e. decreasing price of healthier items) and a combination of both taxation and subsidies in a simulated food delivery app. We found no significant effects of any of the pricing interventions on likelihood of selecting a healthier main meal, total kcal ordered, proportion of healthier *v*. less healthy meal items ordered or nutrients (fat, sugar, salt) selected.

Previous systematic reviews and meta-analyses have shown mixed results on the effects of taxation and subsidies on healthier eating^(15,25,37)^. The studies that found statistically significant effects of pricing interventions using experimental designs tended to implement larger price-based differences (from 20–50 %) and/or were accompanied with a health promotion campaign^(15,25)^. In the present study, we examined the effect of smaller price intervention changes (10 %). This may indicate that taxation and subsidies in a food delivery app setting would need to be considerably larger to impact on food selection behaviour and/or need to be promoted. It may also be the case that communication of price changes (e.g. labelled discounts) would be required to bring about changes in consumer behaviour^(38)^. Therefore, further research examining the effects of different magnitudes of price change with and without advertisement of price changes warrants further investigation, specifically in the context of food delivery services.

Most studies examining food pricing interventions to date have been implemented in supermarkets and workplace/school cafeterias^(15,25)^, as opposed to a food delivery app setting. Food purchasing behaviour may differ in the setting of food delivery services compared to other settings; for instance, it has been shown that demand for main meals is price elastic when in restaurants and (workplace and school) canteens but price may be relatively inelastic in fast-food outlets (which includes takeaway foods)^(39)^. As the use of online food delivery services has grown in recent years^(7)^, and is associated with poorer diet and obesity^(4–6)^, further research to better understand food selection behaviour in food delivery apps will be important.

In our study, demographic characteristics and SEP did not modify the effect of the different pricing interventions on food selection, suggesting that effects of interventions were similar irrespective of participant SEP and other demographics. A recent systematic review, however, reported that with the limited number of studies that reported SEP differences, lower income groups seemed more responsive to taxation of unhealthy foods than higher income groups^(25)^. One possible explanation of the lack of taxation effect in the present study is that irrespective of SEP, consumers consider delivery of out-of-home food to be a ‘treat’ or special occasion,^(40)^ and this may in part explain why we failed to identify any differences by SEP (i.e. lack of price sensitivity).

Results from our follow-up questionnaire indicated that participants in our study favoured subsidies compared to taxation as policy interventions in the OHFS. Similar results were found in a recent more extensive study on public attitudes on taxation in the UK, where 48 % of the participants (*n* 2125) supported taxes on unhealthy foods if they were supplemented with subsidies on healthier foods^(41)^. However, from a policy perspective, taxation generates revenue and may therefore be more sustainable than other nutrition interventions. To offset the potentially regressive nature of taxation, the revenues of taxation may be used to finance subsidies on healthier alternatives^(25)^.

### Strengths and limitations

As discussed, most studies on food pricing interventions to date have examined price differences of 20–50 %, which may not be realistic to implement in real-world conditions. Therefore, a strength of the present study was our use of price changes which would be more consistent with the typically smaller changes in customer facing prices that real-world fiscal policies cause^(26)^. A further strength of the study was that we were powered to detect small-sized statistical effects of pricing interventions on outcomes. It is important to note that we were, however, not powered to detect very small effects. For example, participants in the taxation condition selected 44 kcals less than the control group, which is equivalent to a 3 % reduction of the total kcals selected (*p* = 0·09). If a reliable effect and replicated with a larger sample size, this sized difference in kcals selected could have a small beneficial impact on health at population level. We do however note that the taxation condition was (directionally) less likely to order a healthier menu item than the control condition and the proportion of menu items that were healthier *v*. less healthy in the taxation condition was the same as in the control condition. Therefore, the small directional difference on kcals selected should be interpreted with caution. A further strength of the present research was that we used the UK NPM tool to determine menu item healthiness rather than focusing on single nutrients or food categories. NPMs are now recommended by the WHO for fiscal policies^(10)^, and evidence suggests that dietary patterns as a whole may be more effective in reducing obesity and diet-related diseases than focusing on specific food groups^(17)^. A limitation of the UK NPM tool is that it does not account for portion size (i.e. total energy content) and for use in the present study we modified the tool based on total energy content cut offs by dish type (i.e. main, starters, sides).

This study used a simulated food delivery app, and we therefore measured hypothetical food selection. This is a limitation, and it is unclear whether results would be similar in real-world conditions. For example, pricing interventions may have a greater behavioural impact when people are required to spend their own money. However, most participants in this study agreed that the simulated food delivery app was representative of existing food delivery apps, and previous studies have suggested that virtual methodologies are predictive of real-world behaviour on food purchases^(42)^. Furthermore, a previous study using a similar virtual selection task found evidence that menu item pricing did impact food selection^(43)^. Nonetheless, the simulated nature of our study is a limitation and replication in real-world settings would be valuable to increase confidence in findings. There were data exclusions in the study due to participants not completing the ordering task as instructed and this is a limitation. However, loss of data was relatively low (<10 %) and balanced across conditions.

A further limitation is that our final sample tended to have slightly more participants of higher (*v*. lower) income and education levels when compared to the UK population. We did however find no evidence that findings differed by participant income or education status.

### Implications and conclusions

The use of food delivery platforms has increased in recent years^(7)^ and has been associated with increased risk of obesity^(9)^; it is therefore an important setting for nutrition interventions to improve healthy eating and reduce obesity levels. Fiscal policies have been recommended by the WHO^(10)^ and have been shown effective in a number of settings, including supermarkets^(11,15,44–46)^. However, there is currently an evidence gap on the effectiveness of fiscal policies in the context of food delivery services and how best they could be implemented. Addressing this evidence gap is essential, as consumer behaviour in food delivery apps may differ from behaviour in supermarkets or cafeterias, where most pricing interventions have been tested. This study found no significant effects of 10 % taxation, subsidies and a combination of taxation and subsidies to alter the price of healthier *v*. less healthy menu items on the healthiness of a hypothetical food order compared to standard pricing. However, further research is needed, preferably in the real-world, to understand whether fiscal policies are effective with a larger price difference or in combination with a mass communication campaign (to communicate price differences) or an educational campaign (to communicate the effects of healthier *v*. less healthy foods).

## Supporting information

10.1017/S1368980026103334.sm001 Witkam et al. supplementary material Witkam et al. supplementary material

## Data Availability

Data are available on the Open Science Framework, https://osf.io/ahx8m.

## Supplementary material

For supplementary material accompanying this paper visit https://doi.org/10.1017/S1368980026103334

## Table 1. Long description

The table presents descriptive statistics of a sample (n 3106) across four conditions: Taxation, Subsidies, Combination, and Standard pricing. It includes 18 rows and 8 columns, with column headers indicating the condition and the number of participants (n) and percentage (%). Row labels cover various sample characteristics such as age, sex, ethnicity, income, education, employment status, BMI, and outcome variables like total kcal ordered and total spend. Each row provides mean values, standard deviations, and percentages for each condition. Notable trends include similar mean ages around 37 years, approximately 50% female participants, and around 75% White ethnicity across all conditions. Higher income status is slightly more prevalent, and higher food security is consistent across groups. Employment status varies slightly, with full-time or part-time employment being the most common. BMI and overweight or obesity percentages are also relatively consistent. Outcome variables show minor differences in healthier meal orders, total kcal, fat, saturated fat, salt, sugar ordered, and total spend.

Navigate back to Table 1..

## Table 2. Long description

A table comparing the effects of pricing interventions on meal selection and total kcal selected. The table has two main sections: Healthier v. less healthy main meal selected and Total kcal selected. Each section contains conditions, odds ratios (OR) or regression coefficients, 95% confidence intervals (CI), and p-values. The first section has six rows and four columns, comparing different conditions like taxation, subsidies, and their combination against control and each other. The second section also has six rows and four columns, detailing the regression coefficients, 95% CI, and p-values for the same conditions. Each row provides specific values for OR or regression coefficients, CI, and p-values, showing the effects of the interventions on meal selection and kcal intake.

Navigate back to Table 2..

## Table 3. Long description

A table comparing the effects of pricing interventions (taxation, subsidies, and a combination of both) on various secondary outcomes in a sample size of 3106. The table has 25 rows and 4 columns. Column headers are Conditions, Regression coefficient, 95 percent CI, and p-value. Row labels include different conditions and outcomes such as the proportion of items selected that were healthier, total sugar selected, total fat selected, total saturated fat selected, total salt selected, and total spent. Each row provides regression coefficients, 95 percent confidence intervals, and p-values for different comparisons like Taxation v. control, Subsidies v. control, Combination v. control, and Taxation v. subsidies. Notable trends include varying regression coefficients and confidence intervals across different conditions and outcomes, with most p-values indicating no statistically significant effects.

Navigate back to Table 3..

## Table 4. Long description

The table presents survey responses from participants who completed a follow-up questionnaire. It has four rows and seven columns, with column headers indicating levels of agreement: Strongly disagree, Disagree, Slightly disagree, Neither agree or disagree, Slightly agree, Agree, and Strongly agree. Each row corresponds to a different question. Row 1: I felt that the app was representative of existing food apps. Row 2: To what extent do you agree with the government charging out-of-home food businesses more tax when they sell unhealthy dishes to help people make healthier choices? Row 3: To what extent do you agree with the government charging out-of-home food businesses less tax when they sell healthier dishes to help people make healthier choices? Row 4: To what extent do you agree with the government implementing a combination of taxing businesses more for selling unhealthy dishes whilst taxing businesses less for selling healthier dishes in the out-of-home food sector to help people make healthier choices? Each cell contains the number of responses (n) and the percentage (%) for each level of agreement.

Navigate back to Table 4..
