# Supplementary material for: Socioeconomic position and the effect of price interventions on healthier and less healthy food on food selection in a simulated food delivery app: a randomised controlled trial
Source: Public Health Nutr. 2026 Aug 10;29(1):e188. doi: 10.1017/S1368980026103334 (PMC13740161; doi:10.1017/S1368980026103334)
Supplement: Supplementary file 1 [file S1368980026103334sup001.docx]

## Supplementary materials

Box S1: Detailed criteria for classifying meals as “healthy” vs “less healthy”

Healthiness of mains, sides and desserts was assessed using the UK Nutrient Profile Model (NPM) ^1^. A score was assigned to four “less healthy” components per 100 grams: energy (kJ), saturated fat (g), total sugar (g) and sodium (mg) (Table S1). A score was then assigned to three “healthier” components per 100 grams: fruit, vegetables and nuts (%), fibre (g) and protein (g) (Table S2). The scores for the healthier components were deducted from the scores for the less healthy components to produce an overall NPM score.

If a product scored 11 points or more for the less healthy components, it could only receive protein points if it also scored at least 5 points for fruit, vegetables and nuts. Products with an overall score of 4 or more were classified as less healthy, whereas products scoring below 4 were classified as healthier.

The score for the fruit, vegetables and nuts component was based on the ingredient list when available; if this was not available, a judgement was made using a picture of the dish. To reduce bias, 20% of these pictures were coded by a second researcher. The NPM calculator, developed by researchers at the University of Leeds, was used to calculate the NPM scores of the dishes (<https://npmcalculator.cdrc.ac.uk/>).

As NPM did not account for serving size, main dishes with excessive energy content (defined as 1000 kcal or more by Robinson et al., 2018^2^) were additionally classified as less healthy. Sides and desserts containing 600 kcal or more were similarly classified as less healthy, based on Public Health England recommendations for a complete main meal and the definition of excessive energy content for sides and desserts provided by Muc et al., 2019^3^.

Table S1: Point system for the “less healthy” components per 100 grams

| **Points** | **Energy (kJ)** | **Saturated Fat (g)** | **Total Sugar (g)** | **Sodium (mg)** |
| --- | --- | --- | --- | --- |
| 0 | ≤335 | ≤1 | ≤4.5 | ≤90 |
| 1 | >335 | >1 | >4.5 | >90 |
| 2 | >670 | >2 | >9 | >180 |
| 3 | >1005 | >3 | >13.5 | >270 |
| 4 | >1340 | >4 | >18 | >360 |
| 5 | >1675 | >5 | >22.5 | >450 |
| 6 | >2010 | >6 | >27 | >540 |
| 7 | >2345 | >7 | >31 | >630 |
| 8 | >2680 | >8 | >36 | >720 |
| 9 | >3015 | >9 | >40 | >810 |
| 10 | >3350 | >10 | >45 | >900 |

kJ, kilojoules; g, grams; mg, milligrams.

Table S2: Point system for “healthier” components per 100 grams

| **Points** | **Fruit, Veg & Nuts (%)** | **Fibre (g)** | **Protein (g)** |
| --- | --- | --- | --- |
| 0 | ≤40 | ≤0.7 | ≤1.6 |
| 1 | >40 | >0.7 | >1.6 |
| 2 | >60 | >1.4 | >3.2 |
| 3 | - | >2.1 | >4.8 |
| 4 | - | >2.8 | >6.4 |
| 5 | >80 | >3.5 | >8.0 |

g, grams.

Box S2: Virtual food delivery app – home page

Box S3: Virtual food delivery app – menu page for Japanese restaurant chain

Box S4: Virtual food delivery app – menu page for Mexican fast food

Box S5: Virtual food delivery app – menu page for healthier on the go fast food

Box S6: Virtual food delivery app – menu page for pizza restaurant chain

Box S7: Virtual food delivery app – Click on the basket and inside the basket

To complete the order, participants had to click on the basket and confirm their order, as shown below.

Table S3: The total number and the mean price of mains and proportion of healthier and less healthy mains per outlet

|  | **Mains** | | | |
| --- | --- | --- | --- | --- |
|  | **Total** | **Healthier**  **Mean price, £ (n)** | **less healthy**  **Mean price, £ (n)** | **% Healthier** |
| Mexican fast food | 11 | £7.99 (n=4) | £8.82 (n=7) | 36.4% |
| Healthier on the go fast food | 18 | £7.24 (n=15) | £9.82 (n=3) | 83.3% |
| Japanese restaurant chain | 40 | £13.42 (n=25) | £13.93 (n=15) | 62.5% |
| Pizza restaurant chain | 47 | £14.52 (n=20) | £15.11 (n=27) | 42.6% |
| *Total* | *116* | *64* | *52* | *55.2%* |

Table S4: The total number and the mean price of sides and proportion of healthier sides per outlet

|  | **Sides** | | | |
| --- | --- | --- | --- | --- |
|  | **Total** | **Healthier**  **Mean price, £ (n)** | **Less healthy**  **Mean price, £ (n)** | **% Healthier** |
| Mexican fast food | 10 | £1.49 (n=1) | £4.22 (n=9) | 10.0% |
| Healthier on the go fast food | 12 | £3.53 (n=8) | £6.92 (n=4) | 66.7% |
| Japanese restaurant chain | 17 | £6.71 (n=10) | £7.71 (n=7) | 58.8% |
| Pizza restaurant chain | 14 | £5.55 (n=5) | £7.36 (n=9) | 35.7% |
| *Total* | *53* | *24* | *29* | *45.3%* |

Table S5: The total number and the mean price of desserts and proportion of healthier desserts per outlet

|  | **Desserts** | | | |
| --- | --- | --- | --- | --- |
|  | **Total** | **Healthier**  **Mean price, £ (n)** | **Less healthy**  **Mean price, £ (n)** | **% Healthier** |
| Mexican fast food | 5 | 0 | £3.99 (n=5) | 0.0% |
| Healthier on the go fast food | 9 | 0 | £2.96 (n=9) | 0.0% |
| Japanese restaurant chain | 1 | 0 | £7.50 (n=1) | 0.0% |
| Pizza restaurant chain | 6 | 0 | £7.45 (n=6) | 0.0% |
| *Total* | *21* | *0* | *21* | 0.0% |

Box S8: Detailed sample size calculation

G*power was used for sample size calculations for the primary analysis. To estimate the required sample sizes for the interventions compared to control, a previous meta-analysis was used that found that a 10% decrease in price was associated with increased consumption of healthy products by 12%^4^.

The baseline odds (i.e. H0) of choosing healthier dishes is expected to be 0.55 (based on the proportion of healthier vs less healthy main meals at the four included restaurants, and the expectation that each dish has the same chance to be selected, see Table 1). The odds of choosing healthier dishes in the intervention group of the 10% price decrease was expected to be 0.62 (0.55*1.12, i.e. H0*12% increase in consumption of healthier products). This leads to an odds ratio of 1.33. We used a two-tail test, the power was set at 80% and the alpha at 5%. R-squared was set at 0 as any covariates (e.g. SEP) are not expected to be associated with the variability of the main predictor variable (i.e. intervention group), as participants are randomised in the four different groups and thus any covariates are balanced within those groups. The X parameter is set at 0.5, as the proportion of people in the intervention group vs control is equal. Based on these parameters, the estimated total participants needed to compare a price decrease of 10% to the control group would be 1557 (i.e. 779 in the price decrease group and 779 in the control group). We used the same reasoning to determine number of participants in the other two intervention conditions, resulting in a total of N=3116.

We were further interested in understanding whether the effectiveness differs for participants with lower versus higher SEP. A study in New Zealand estimated price elasticities for different food groups by income quintiles^5^. They found that for “ready to eat foods” (i.e. takeaway foods), price elasticities were -2.1 for the lowest income quintile and -0.01 for the highest income quintile. This means that with a 10% increase in price, consumption of ready to eat foods may decrease by 21% in the lowest income quintile and by 0.1% in the highest income quintile. This indicates that for the highest income quintile, the price for ready to eat foods at home was inelastic in this study.

However, we were able to use this study’s price elasticity for low-income households to estimate the sample size needed to find an effect for a 10% price increase on less healthy foods in a takeaway setting for low-income households. The baseline odds of buying less healthy foods was 0.45 (i.e. based on the proportion of less healthy foods in our study). The odds of buying less healthy foods with a 10% price increase would be 0.35 (0.45*0.79, i.e. 0.45*a reduction of 21%). This translates to an OR of 0.66 for buying less healthy foods with the 10% price increase compared to no price increase. Using the same estimates in G*power as before, a sample size of 594 is needed to find the effect of a 10% price increase for low income households (i.e. 297 for the intervention group and 297 for the control group). As we aim to recruit above this number of low SEP participants we will therefore be powered to detect a moderate sized SEP interaction and detect anticipated effects of pricing intervention in low SEP participants (in follow-up stratified analysis).

Because there is uncertainty in expected effects and there will likely be a small amount of data loss (e.g., participants guessing aims failing attention checks), if feasible within the study timeframe, we will aim to recruit up to an additional 10%. This leads to a sample size of 3,464 (i.e. 866 participants per group).

Box S9: Minor food ordering errors

There were n=254 participants (8.2% of the total sample) who had duplicate orders. These were evenly spread among the different conditions. Duplicates were most common for mains (n=145), followed by drinks (n=85), sides (n=71) and desserts (n=38). After careful consideration, we concluded that the duplicates were errors and did not reflect an order that would be made in the real-world (for instance, some participants ordered the same pizza five times). We therefore only recorded the first item in the duplicated order.

Table S6: The effect of the pricing interventions (taxation, subsidies and a combination of taxation and subsidies) on primary outcomes in final sample by income status (n=3106)

|  | **Higher income*** | | **Lower income**† | |
| --- | --- | --- | --- | --- |
| **Healthier vs less healthy main meal ordered** | | | | |
| **Conditions** | **OR (95% CI)** | **p-value** | **OR (95% CI)** | **p-value** |
| Taxation vs control | 0.87 (0.66, 1.14) | 0.32 | 0.95 (0.69, 1.31) | 0.76 |
| Subsidies vs control | 1.12 (0.86, 1.45) | 0.41 | 1.00 (0.73, 1.37) | 0.99 |
| Combination vs control | 1.09 (0.84, 1.43) | 0.52 | 0.94 (0.69, 1.29) | 0.69 |
| Taxation vs subsidies | 0.78 (0.60, 1.02) | 0.07 | 0.95 (0.70, 1.30) | 0.75 |
| **Total kcal purchased** | | | | |
| **Conditions** | **Regression coefficient (95% CI)** | **p-value** | **Regression coefficient (95% CI)** | **p-value** |
| Taxation vs control | -45.75 (-116.02, 24.52) | 0.20 | -49.13 (-129.77, 31.50) | 0.23 |
| Subsidies vs control | -12.88 (-82.65, 56.88) | 0.72 | -26.21 (-110.04, 57.61) | 0.54 |
| Combination vs control | -22.14 (-91.73, 47.45) | 0.53 | -28.73 (-115.22, 57.76) | 0.51 |
| Taxation vs subsidies | -32.87 (-101.93, 36.19) | 0.35 | -22.92 (-99.43, 53.60) | 0.56 |

*Defined as a monthly net household income of higher than £1500. †Defined as a monthly net household income of £1500 or lower. CI, confidence interval; OR, odds ratio.

Table S7: The effect of the pricing interventions (taxation, subsidies and a combination of taxation and subsidies) on secondary outcomes in final sample by income status (n=3106)

|  | **Higher income*** | | **Lower income**† | |
| --- | --- | --- | --- | --- |
| **Conditions** | **Regression coefficient (95% CI)** | **p-value** | **Regression coefficient (95% CI)** | **p-value** |
| **Proportion of healthier meal items vs less healthy meal items purchased** | | | | |
| Taxation vs control | 0.01 (-0.20, 0.23) | 0.91 | -0.03 (-0.29, 0.23) | 0.84 |
| Subsidies vs control | 0.05 (-0.16, 0.26) | 0.65 | 0.02 (-0.23, 0.28) | 0.86 |
| Combination vs control | 0.08 (-0.14, 0.29) | 0.48 | -0.00 (-0.26, 0.25) | 0.97 |
| Taxation vs subsidies | -0.04 (-0.25, 0.18) | 0.74 | -0.05 (-0.30, 0.20) | 0.70 |
| **Total sugar purchased** | | | | |
| Taxation vs control | 0.18 (-2.86, 3.23) | 0.91 | -1.53 (-5.30, 2.24) | 0.43 |
| Subsidies vs control | -0.99 (-3.99, 2.01) | 0.52 | -0.78 (-4.51, 2.95) | 0.68 |
| Combination vs control | 0.97 (-2.16, 4.09) | 0.54 | -1.94 (-5.46, 1.57) | 0.28 |
| Taxation vs subsidies | 1.17 (-1.92, 4.27) | 0.46 | -0.76 (-4.48, 2.97) | 0.69 |
| **Total fat purchased** | | | | |
| Taxation vs control | -2.03 (-5.61, 1.54) | 0.27 | -2.53 (-6.59, 1.54) | 0.22 |
| Subsidies vs control | -0.40 (-3.83, 3.03) | 0.82 | -1.31 (-5.41, 2.80) | 0.53 |
| Combination vs control | -0.20 (-3.70, 3.30) | 0.91 | -1.20 (-5.52, 3.12) | 0.59 |
| Taxation vs subsidies | -1.63 (-5.07, 1.81) | 0.35 | -1.22 (-5.04, 2.61) | 0.53 |
| **Total saturated fat purchased** | | | | |
| Taxation vs control | -0.11 (-1.57, 1.34) | 0.88 | -0.69 (-2.28, 0.90) | 0.40 |
| Subsidies vs control | 0.11 (-1.31, 1.52) | 0.88 | -0.62 (-2.27, 1.04) | 0.47 |
| Combination vs control | -0.07 (-1.50, 1.37) | 0.93 | 0.17 (-1.56, 1.90) | 0.85 |
| Taxation vs subsidies | -0.22 (-1.65, 1.20) | 0.76 | -0.07 (-1.69, 1.54) | 0.93 |
| **Total salt purchased** | | | | |
| Taxation vs control | -0.16 (-0.60, 0.28) | 0.48 | -0.04 (-0.54, 0.45) | 0.86 |
| Subsidies vs control | -0.07 (-0.53, 0.38) | 0.76 | 0.15 (-0.37, 0.68) | 0.57 |
| Combination vs control | -0.32 (-0.77, 0.13) | 0.17 | 0.09 (-0.46, 0.64) | 0.74 |
| Taxation vs subsidies | -0.09 (-0.54, 0.36) | 0.70 | -0.20 (-0.67, 0.28) | 0.41 |
| **Total spend** | | | | |
| Taxation vs control | 0.50 (-0.86, 1.86) | 0.47 | 0.20 (-1.33, 1.73) | 0.80 |
| Subsidies vs control | -1.11 (-2.44, 0.22) | 0.10 | -0.37 (-1.95, 1.21) | 0.65 |
| Combination vs control | -0.19 (-1.57, 1.18) | 0.78 | 0.04 (-1.57, 1.66) | 0.96 |
| Taxation vs subsidies | 1.61 (0.27, 2.96) | 0.02 | 0.57 (-0.91, 2.04) | 0.45 |

*Defined as a monthly net household income of higher than £1500. †Defined as a monthly net household income of £1500 or lower. CI, confidence interval.

Table S8: The effect of the pricing interventions (taxation, subsidies and a combination of taxation and subsidies) on primary outcomes in a sub sample (i.e. those with dietary restrictions / food allergies were excluded) (n=2705)

| *Healthier vs less healthy main meal ordered* | | |
| --- | --- | --- |
| **Conditions** | **OR (95% CI)** | **p-value** |
| Taxation vs control | 0.88 (0.71, 1.09) | 0.24 |
| Subsidies vs control | 1.05 (0.85, 1.30) | 0.63 |
| Combination vs control | 0.96 (0.77, 1.18) | 0.68 |
| Taxation vs subsidies | 0.83 (0.67, 1.03) | 0.10 |
| *Total kcal purchased* | | |
| **Conditions** | **Regression coefficient (95% CI)** | **p-value** |
| Taxation vs control | -32.0 (-87.2, 23.2) | 0.26 |
| Subsidies vs control | 8.8 (-48.0, 65.7) | 0.76 |
| Combination vs control | -21.2 (-77.0, 34.6) | 0.46 |
| Taxation vs subsidies | -40.8 (-95.7, 14.1) | 0.15 |

CI, confidence interval; OR, odds ratio.

Table S9: The effect of the pricing interventions (taxation, subsidies and a combination of taxation and subsidies) on primary outcomes in those who did not guess the study aim (n=2863)

| *Healthier vs less healthy main meal ordered* | | |
| --- | --- | --- |
| **Conditions** | **OR (95% CI)** | **p-value** |
| Taxation vs control | 0.90 (0.73, 1.12) | 0.35 |
| Subsidies vs control | 1.07 (0.87, 1.31) | 0.54 |
| Combination vs control | 1.02 (0.83, 1.26) | 0.86 |
| Taxation vs subsidies | 0.85 (0.69, 1.04) | 0.12 |
| *Total kcal purchased* | | |
| **Conditions** | **Regression coefficient (95% CI)** | **p-value** |
| Taxation vs control | -45.48 (-98.91, 7.96) | 0.10 |
| Subsidies vs control | -1.70 (-56.05, 52.64) | 0.95 |
| Combination vs control | -22.32 (-77.00, 32.36) | 0.42 |
| Taxation vs subsidies | -43.77 (-97.00, 9.46) | 0.11 |

CI, confidence interval; OR, odds ratio.

Table S10: Interaction analysis of socioeconomic and demographic variables on the primary outcomes for each condition

|  | Healthier vs less healthy  (p-value) | Total kcal  (p-value) |
| --- | --- | --- |
| **Income (mean-centered*)** | | |
| Taxation vs control | 0.24 | 0.15 |
| Subsidies vs control | 0.80 | 0.65 |
| Combination vs control | 0.88 | 0.28 |
| Taxation vs subsidies | 0.34 | 0.31 |
| **Household food security (high vs low)** | | |
| Taxation vs control | 0.56 | 0.60 |
| Subsidies vs control | 0.14 | 0.38 |
| Combination vs control | 0.77 | 0.83 |
| Taxation vs subsidies | 0.04 | 0.15 |
| **Education (high vs low)** | | |
| Taxation vs control | 0.37 | 0.66 |
| Subsidies vs control | 0.95 | 0.83 |
| Combination vs control | 0.49 | 0.32 |
| Taxation vs subsidies | 0.33 | 0.50 |
| **Subjective socioeconomic status (mean-centered)** | | |
| Taxation vs control | 0.63 | 0.71 |
| Subsidies vs control | 0.39 | 0.54 |
| Combination vs control | 0.65 | 0.26 |
| Taxation vs subsidies | 0.73 | 0.80 |
| **Sex (female vs male)** | | |
| Taxation vs control | 0.98 | 0.88 |
| Subsidies vs control | 0.11 | 0.39 |
| Combination vs control | 0.17 | >0.99 |
| Taxation vs subsidies | 0.12 | 0.47 |
| **BMI (mean-centered)** | | |
| Taxation vs control | 0.05 | 0.26 |
| Subsidies vs control | 0.56 | 0.78 |
| Combination vs control | 0.76 | 0.69 |
| Taxation vs subsidies | 0.15 | 0.15 |
| **Ethnic group (white vs non-white)** | | |
| Taxation vs control | 0.35 | 0.28 |
| Subsidies vs control | 0.48 | 0.96 |
| Combination vs control | 0.17 | 0.50 |
| Taxation vs subsidies | 0.80 | 0.29 |

*Continuous variables were mean-centered to reduce multi-collinearity.

**References**

1.	Care DoHaS. Nutrient Profiling Technical Guidance. 2011.

2.	Robinson E, Jones A, Whitelock V, Mead BR, Haynes A. (Over)eating out at major UK restaurant chains: observational study of energy content of main meals. BMJ (Clinical research ed). 2018;363:k4982.

3.	Muc M, Jones A, Roberts C, Sheen F, Haynes A, Robinson E. A bit or a lot on the side? Observational study of the energy content of starters, sides and desserts in major UK restaurant chains. BMJ open. 2019;9(10):e029679.

4.	Afshin A, Peñalvo JL, Del Gobbo L, Silva J, Michaelson M, O'Flaherty M, et al. The prospective impact of food pricing on improving dietary consumption: A systematic review and meta-analysis. PloS one. 2017;12(3):e0172277.

5.	Ni Mhurchu C, Eyles H, Schilling C, Yang Q, Kaye-Blake W, Genç M, et al. Food prices and consumer demand: differences across income levels and ethnic groups. PLoS One. 2013;8(10):e75934.
